# Stage-specific exposure of *Caenorhabditis elegans* to cadmium identifies unique transcriptomic response cascades and an uncharacterized cadmium responsive transcript

**DOI:** 10.1093/mtomcs/mfae016

**Published:** 2024-03-28

**Authors:** Norah Almutairi, Naema Khan, Alexandra Harrison-Smith, Volker M Arlt, Stephen R Stürzenbaum

**Affiliations:** Department of Analytical, Environmental and Forensic Sciences, Faculty of Life Sciences and Medicine, King's College London, London, UK; Department of Analytical, Environmental and Forensic Sciences, Faculty of Life Sciences and Medicine, King's College London, London, UK; Department of Analytical, Environmental and Forensic Sciences, Faculty of Life Sciences and Medicine, King's College London, London, UK; Department of Analytical, Environmental and Forensic Sciences, Faculty of Life Sciences and Medicine, King's College London, London, UK; Department of Analytical, Environmental and Forensic Sciences, Faculty of Life Sciences and Medicine, King's College London, London, UK

**Keywords:** *C. elegans*, L3 stage, L4 stage, cadmium, transcriptomics, metallothionein

## Abstract

Age/stage sensitivity is considered a significant factor in toxicity assessments. Previous studies investigated cadmium (Cd) toxicosis in *Caenorhabditis elegans*, and a plethora of metal-responsive genes/proteins have been identified and characterized in fine detail; however, most of these studies neglected age sensitivity and stage-specific response to toxicants at the molecular level. This present study compared the transcriptome response between *C. elegans* L3 vs L4 larvae exposed to 20 µM Cd to explore the transcriptional hallmarks of stage sensitivity. The results showed that the transcriptome of the L3 stage, despite being exposed to Cd for a shorter period, was more affected than the L4 stage, as demonstrated by differences in transcriptional changes and magnitude of induction. Additionally, *T08G5.1*, a hitherto uncharacterized gene located upstream of metallothionein (*mtl-2*), was transcriptionally hyperresponsive to Cd exposure. Deletion of one or both metallothioneins (*mtl-1* and/or *mtl-2*) increased *T08G5.1* expression, suggesting that its expression is linked to the loss of metallothionein. The generation of an extrachromosomal transgene (P*T08G5.1*:: GFP) revealed that *T08G5.1* is constitutively expressed in the head neurons and induced in gut cells upon Cd exposure, not unlike *mtl-1* and *mtl-2.* The low abundance of cysteine residues in *T08G5.1* suggests, however, that it may not be involved directly in Cd sequestration to limit its toxicity like metallothionein, but might be associated with a parallel pathway, possibly an oxidative stress response.

## Graphical Abstract

**Graphical Abstract:**
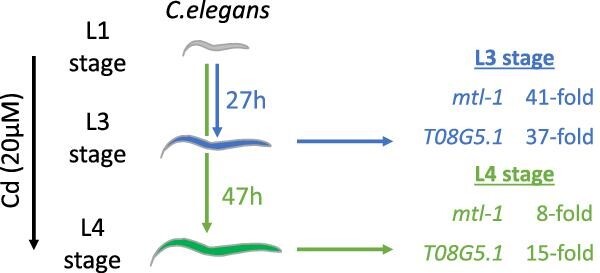
*C. elegans* were exposed from L1 to L3 or L4 stage to identify development-specific changes of the transcriptome.

## Introduction

Cadmium (Cd) is utilized in medium to large scale industrial processes but also in many day-to-day products such as batteries, pigments, coating, and fertilisers.^1,2^ Cd (II) is of toxicological concern as it resembles the chemical properties of Zn (II), and as a softer Lewis acid is capable of binding to ligands to form coordination compounds.^3^ Several studies have described the damage caused by exposure to Cd; for instance, it disturbs the redox balance by displacing essential elements in the cell, which in turn causes oxidative stress, inflammatory responses, and genotoxicity.^4^ The severity of Cd exposure varies depending on various factors, including species, stage sensitivity, concentration, and exposure duration, all of which influence how an organism can cope with the toxic challenge. Several studies have suggested that these toxic substances generally exert more significant effects on early life stages than late(r) life stages.^5^ Hence, guidelines in toxicology testing take into consideration an organism's age/stage.^6^ Stage sensitivity can occur due to physiological, morphological, and biochemical aspects e.g. the reduced size of the animal, the immaturity of an organ, or an underdeveloped detoxification pathway. The majority of ecotoxicological data has been derived from aquatic tests designed to take these aspects into account.^7–11^ To date, significantly less is known regarding the age sensitivity/stage-specific response dynamics in organisms within the terrestrial ecosystem at the molecular level.

The nematode *Caenorhabditis elegans* is a terrestrial organism amenable to toxicity assessments, including heavy metals.^12,13^ It is small in size and has a rapid life cycle. *Caenorhabditis elegans*, when cultured under optimal conditions (20°C and fed ad libitum), goes through four larval stages (L1–L4) and develops into an adult within 3 days. Each larval stage has distinct characteristics, e.g. at L1 stage the neuronal system develops, at L2 and L3 stages the nematode grows in size, and by the end of the L3 stage, the majority of organs are fully developed (but exclude the majority of the reproductive system). Vulva development commences at late L3 stage and the reproductive organs are fully developed by the end of L4 stage.^14^ Further, since nematode is a self-fertilizing hermaphrodite, the worm population is essentially clonal; hence, reduces intra-species variability based on genetic drift. These traits make *C.elegans* a preferable model for environmental toxicology assessment. In addition, the completion of the *C. elegans* genome sequencing project in 1998 revealed the chromosomal location of ∼20 000 protein-coding genes.^15^ The vast majority of these genes are categorized and annotated in fine detail, information, which can be retrieved from the Wormbase database, however, over 20% of genes have no known function and are defined as uncharacterized.^16^  *Caenorhabditis elegans*’ response to Cd exposure is well documented; e.g. Cd was shown to reduce reproductive performance and lifespan, alter cellular structure, modulate gene expression, and induce metabolic changes.^17–26^ These effects were observed in age synchronized populations as well as mixed stages of worms. The sensitivity of early larval stages (L1–L3 stage) in comparison to later stages (L4 stage and young adults) in response to single and/or binary exposure(s) to heavy metals revealed stage-specific differences in physiological parameters, including reproductive capability, and neuro-behavioural changes.^27–29^

Ample data are available concerning the assessment of Cd toxicosis in *C.elegans*; however, to the best of our knowledge, no data have been forthcoming that compares the transcriptomic response to Cd exposure at defined larval life stages. Therefore, the aim of this work is to identify holistic differences that define stage-specific responses and sensitivities to toxicity between early life and late life larval stages L3 and L4, in addition to pinpointing novel targets linked to Cd detoxification and toxicosis.

## Materials and methods

### Experiment design

*Caenorhabditis elegans* (wild-type (WT), N2 Bristol), *mtl-2*(gk125), and *Escherichia coli* (OP50) were obtained from the *Caenorhabditis* Genetics Center (CGC). The *mtl-1*(tm1770) was obtained by the National Bioresource Project headed by the Mitani lab, Tokyo, Japan, and the metallothionein double knockout strain *mtl-1; mtl-2*(zs1) was generated by Dr Sam Hughes (in the Stürzenbaum lab). In addition, a P*T08G5.1*:: GFP strain was generated as part of this study via the microinjection of extrachromosomal transgenic arrays. The standard method of Brenner,^30^ was used to culture synchronized *C. elegans* on Nematode Growth Media (NGM) plates at 20°C, which were inoculated with *E. coli* with or without supplementation of 10, 20, and 40 μM cadmium chloride (Sigma-Aldrich 99% ACS—CdCl_2_). Synchronized L1 stage nematodes were grown either for 27 h to L3 or for 47 h to L4 stage, then collected from the plates using M9 buffer, washed multiple times to remove the bacteria, and then prepared for total RNA extraction using the Zymo Quick-RNA™ kit according to the manufacturer's protocol.

### RNA sequencing

Following the sample generation, collection, and RNA extraction, mRNA was isolated via poly-A selection and RNAseq performed by Illumina HiSeq 150 bp paired-end sequencing (GENEWIZ^©^). Reads (∼40 million reads per replicate) were mapped to the ENSEMBL (WBcel235 version) *Caenorhabditis elegans* reference genome using the STAR aligner (v.2.5.2b). Gene hit counts were calculated (by applying featureCounts from the Subread package, v.1.5.2) and used for downstream differential expression analyses. DESeq2 software identified the variance between tested samples. In order to focus only on the most significant transcripts, p-values and log2 fold cut-offs were applied. The RNAseq data can be accessed via GEO accession GSE1156507.

### Quantitative PCR (qPCR)

Quantitative PCR (qPCR, ABI Prism 7500 Fast System, Applied BioSystems, Warrington, UK) was used to validate the most responsive transcripts by means of TaqMan^®^ and SYBR™ Green assays. The resultant data were then analysed further for functional enrichment using annotation software. Relative gene expression was calculated using the 2^− ΔΔCT^ method, and the resultant C_T_ values normalized to the invariant house-keeping gene *rla-1*.^31^ Data were expressed as mean ± standard error of the mean (SEM), *N* = 3 biological replicates per condition.

### Gene expression statistical analysis

The Wald test was used to generate p-values and log2 fold changes of the RNAseq data. Genes with an adjusted p-value ≤0.05 and absolute log2 fold change ≥1 were defined as differentially expressed genes. Statistically significant data from qPCR was expressed as mean ±SEM, with *t*-test and analysis of variance (ANOVA) statistical analyses performed to determine the significance between conditions.

### Transgenic worms

The promoter sequence (580 bp) of *T08G5.1* was synthesized (ThermoFisher^®)^, cloned in frame into the GFP vector *pPD95_75*, and then injected with the marker *rol-6* into the gonads of *C. elegans*. The microinjection process was performed by SunyBiotech^®^, China. Transgenic worms were synchronized and seeded in the presence or absence of Cd. Transgenic worms were observed under an inverted microscope (Nikon ECLIPSE TE2000-S) with blue laser scanning fluorescence and/or a confocal microscope (Nikon A1R) at *λ*_ex_ = 450–490 nm. Images were taken with the camera attached to the microscope and analysed utilizing ImageJ 1.49C software.

### RNA interference of *T08G5.1*

To facilitate the characterization of *T08G5.1*, knockdown by RNA interference was performed. First, RNAi NGM plates were prepared containing ampicillin (100 µg/ml) and isopropyl β-d-1-thiogalactopyranoside (1 mM IPTG). The plates were maintained at room temperature for at least 2 days prior to inoculating them with the culture of bacteria expressing dsRNA as specified by Timmons *et al*.^32^ The HT115 bacteria that carry the double T7 RNAi vector (L4440) with *T08G5.1* inserted into the multiple cloning site were streaked onto an NGM plate supplemented with ampicillin (100 µg/ml) and incubated overnight at 37°C. Subsequently, a single bacterial colony was inoculated into LB broth containing 100 µg/ml ampicillin and incubated for 6–8 h at 37°C in a shaking incubator. Thereafter, the fresh bacterial culture (200 µl) was seeded onto the NGM RNAi plates (containing ampicillin and IPTG), then incubated for 2 days at room temperature in a dark box. Finally, synchronized larva (L1) worms were seeded onto the RNAi plates and allowed to grow for at least two generations to facilitate gene silencing. RNAi *T08G5.1* worms were then cultured either in HT115 that carry the vector of dsRNA (RNAi *T08G5.1*) or in HT115, which contained the empty vector (RNAi control) in the presence of absence of cadmium chloride. The efficiency of the knockdown was examined by means of qPCR.

#### Reproduction assay

The reproduction assay was conducted on WT worms and RNAi worms, by allowing synchronized L1 to grow (in the presence or absence of Cd) either to L3 stage or to L4 stage. At the end of the exposure period, the worms were collected, washed multiple times, and transferred individually to 12 well NGM plates inoculated with OP50 or HT115 for RNAi, typically 10 replicates per condition. Worms were transferred to a new well every 24 h until reproduction ceased. The daily and total number of hatched progenies were determined by observation and manual counting.

## Results and discussion

### Concentration validation by reproduction assay

**Fig. 1.**
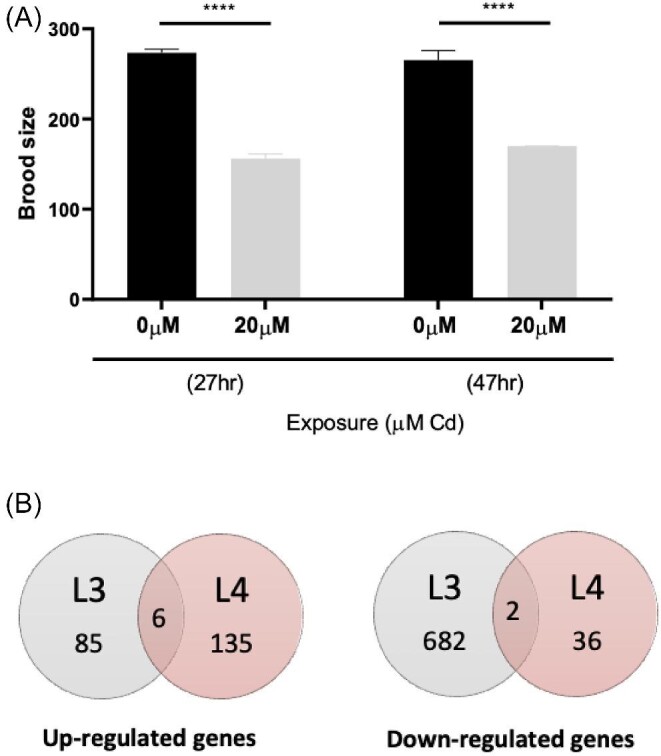
Reproductive performance in synchronized WT *C. elegans* grown in the presence or absence of 20 µM Cd. Worms were exposed from L1 to L3 (27 h) and L1–L4 developmental stage (47 h). Statistical difference in total reproductive output was determined by two-way ANOVA followed by a Sidak's multiple comparisons test (*P ≤ 0.05, **P ≤ 0.01, and ***P ≤ 0.0001), values represent mean ± SEM (*n* = 10). Note: The total brood size decreased in Cd exposed worms in comparison to the untreated conditions, but this was independent of exposure length and stage (**A**). Venn diagrams showing the number of significantly changing genes at the L3 and L4 stage following an exposure to 20 µM Cd, the selection criteria included a fold-change of ≥ 2 or ≤ −2 and a *P*-value of ≤ 0.05. Only six transcripts were commonly overexpressed in the L3 and L4 stage, namely *mtl-1, mtl-2, cdr-1, lipl-1, F56C3.9*, and *T08G5.1. F09C8.1* and *cest-1* were the only two transcripts that were down-regulated at both stages (**B**).

Exposure to Cd during the larval stages resulted in a large reduction in offspring produced once the worms developed to adults. This was independent of exposure duration, namely from L1 to L3 stage (27 h exposure) or L1–L4 stage (47 h exposure) (Fig. 1-A). The analysis aligns with the published literature, which suggests that Cd interferes with the homeostatic control of zinc, copper, and calcium, namely elements that are required for the normal development of the organism.^24,33–36^ In *C. elegans*, Zn is required for sperm activation, which is mediated specifically by the ZIPT-7.1 transporter, as well as the maturation of the oocyte. These two observations may explain why a low dose of Cd during the larval development can lead to a significant reduction in reproductive performance, regardless of exposure duration.^37–39^

### Transcriptome analysis of the cadmium exposome

Since the exposure to the sub-lethal concentration of Cd led to growth impairment in both L3 and L4, this research further explored the notion that stage-specific differences in response profiles drive the general response to Cd toxicosis. The analysis revealed a set of 775 differentially expressed transcripts in worms exposed from the L1 stage to the L3 stage, a number that was significantly higher than the 179 differentially expressed genes observed in worms exposed from L1 to L4 stage. At the L3 stage, the majority (88%) of the differentially expressed transcripts were down-regulated, but the opposite occurred at the L4 stage, where 79% of the significantly changing transcripts were up-regulated (Fig. 1-B).

Some significantly up-regulated transcripts were common to both stages. Whilst three of those genes were previously identified as Cd/metal responsive genes, namely *mtl-1, mtl-2, and cdr-*1, others were not. For example, *lipl-4*, which is linked to lipid metabolism and *T08G5.1*, an uncharacterized gene with no annotated function. There was no overlap between the two stages in terms of the most down-regulated transcripts (Table 1). Worms exposed from L1 to L3 stage exhibited, in general, a more dynamic response range than worms exposed from L1 to L4 stage (as reflected by the L3’s higher fold change values in both the most up- or down-regulated transcripts), suggesting that the L3 stage, despite being exposed for a shorter time, is transcriptionally more active than the L4 stage. To what extend the interplay between exposure duration and developmental stage affects the transcriptional landscape still needs to be untangled.

**Table 1.** Differential gene expression of *C. elegans*.

A						
	**L3 stage**			**L4 stage**		
Rank	Gene	Fold change	*P*-value	Gene	Fold change	*P*-value
1	*mtl-1*	41.1 ± 2	0	*T08G5.1*	14.5 ± 5	6.4E-18
2	*T08G5.1*	37.4 ± 6	3.8E-11	*mtl-1*	7.8 ± 2	2.1E-21
3	*K04A8.20*	33.0 ± 42	1.3E-15	*lipl-4*	6.5 ± 7	3.01E-07
4	*cdr-1*	10.6 ± 0.1	1.2E-15	*C49G7.7*	4.7 ± 6	1.01E-05
5	*lipl-4*	8.7 ± 2	7.7E-27	*fipr-26*	4.6 ± 1	0.0007
6	*T20F10.9*	8.2 ± 2	0.006	*Y53F4B.8*	4.4 ± 0.7	1.02E-16
7	*pho-10*	7.2 ± 3	1.7E-13	*mtl-2*	4.3 ± 2	0.0006
8	*F56C3.9*	6.2 ± 0.2	2.6E-40	*cdr-1*	4.0 ± 1	2.5E-08
9	*F44E7.5*	5.6 ± 0.1	4.03E-10	*W03F9.4*	3.7 ± 1	0.03
10	*cyp-13A4*	3.9 ± 4	0.004	*C06B3.7*	3.7 ± 1	1.3E-15
11	*mtl-2*	3.6 ± 0.9	2.9E-20	*K09D9.1*	3.5 ± 1	2.4E-08
12	*hrg-2*	3.6 ± 2	0.0001	*C45B11.2*	3.4 ± 1	0.001
13	*C05G5.8*	4.9 ± 2	0.01	*F56C3.9*	3.2 ± 0.9	8.2E-11
14	*acs-2*	3.1 ± 2	1.02E-09	*irg-2*	3.2 ± 0.6	0.0001
15	*zipt-2.3*	2.9	3.7E-08	*F20B10.3*	3.0 ± 0.7	0.0004
**B**						
	**L3 stage**			**L4 stage**		
Rank	Gene	Fold change	*P*-value	Gene	Fold change	*P*-value
1	*F23F1.2*	−10.4 ± 0.04	2.3E-13	*F09C8.1*	−4.6 ± 0.2	0.04
2	*C06A1.8*	−9.0 ± 0.5	0.03	*ZK218.5*	-4.2 ± 0.3	0.004
3	*M01B2.8*	−8.5 ± 0.01	1.1E-06	*clec-47*	−4.0 ± 0.1	4.9E-08
4	*oac-30*	−8.4 ± 0.009	2.8E-13	*K06H6.2*	−3.4 ± 0.3	0.01
5	*Y52B11B.1*	−8.3 ± 0.07	3.9E-08	*math-45*	−3.2 ± 0.1	0.001
6	*Y73B6BL.37*	−8.4 ± 0.08	1.8E-05	*nspc-16*	−3.0 ± 0.7	0.04
7	*K02E11.10*	−7.8 ± 0.05	1.8E-15	*gmd-2*	−2.9 ± 0.3	0.03
8	*C27D9.2*	−7.7 ± 0.07	4.7E-11	*Y48E1B.8*	−2.6 ± 0.2	0.003
9	*clec-229*	−7.6 ± 0.05	6.5E-09	*T24C2.2*	−2.5 ± 0.2	0.02
10	*K01D12.9*	−7.6 ± 0.07	2.2E-13	*ugt-63*	−2.5 ± 0.2	0.002
11	*R12E2.15*	−7.5 ± 0.06	5.3E-13	*cest-1*	−2.4 ± 0.1	0.04
12	*wrt-8*	−7.2 ± 0.04	4.1E-11	*ccch-5*	−2.4 ± 0.1	0.008
13	*W02F12.8*	−7.1 ± 0.04	6.6E-20	*Y22D7AR.10*	−2.4 ± 0.1	2.9E-05
14	*F45B8.3*	−7.1 ± 0.09	3.0E-05	*F15E6.4*	−2.4 ± 0.1	0.005
15	*lfor-1*	−6.9 ± 0.2	0.004	*skr-15*	−2.3 ± 0.1	0.01
	*….*	….	….			
	*cest-1*	−3.7 ± 0.1	1.7E-10			
	*F09C8.1*	−2.3 ± 0.06	6.8E-08			

Nematodes were treated with 20 µM Cd from L1 to L3 stage (27 h) or from L1 to L4 stage (47 h). (**A**) The most highly expressed genes with a *P*-value of ≤ 0.05 are listed. Note: *mtl-1, mtl-2, cdr-1, lipl-4, F56C3.9*, and *T08G5.1* are common to both lists. (**B**) The most down-regulated gene with a *P*-value of ≤ 0.05 are listed. Note: only *F09C8.1* and *cest-1* were common to both stages. The transcripts common to both stages are underlined.

To provide a means of data validation, a subsection of the most overexpressed genes (*mtl-1, cdr-1, T08G5.1, K04A8.20, C49G7.7*, and *lipl-4*) were analysed by qPCR (Supplementary Fig. 1). The results confirmed the general trends that were identified by RNAseq, namely that *mtl-1* was highly induced (41.1-fold) in Cd exposed worms at the L3 stage but only 7.7-fold in L4 (compared with their unexposed counterparts). Likewise, the expression of *T08G5.1* increased 37.4 fold in worms exposed from L1 to L3 and 14.5-fold in worms exposed to L4 stage. The most down-regulated transcripts of the RNAseq and qPCR output were also well aligned (data not shown); however, *lipl-4* expression was below the detection limit of the qPCR. The independent validation of the RNAseq results, therefore, provided confidence in the reproducibility of the RNAseq output. To what extent homo- and hetero-mutants attenuate the biological response under Cd exposure is, at this point, not known and worthy of further investigations. Taken together, the findings confirmed that the magnitude of gene induction (i.e. fold-change) was markedly higher at the L3 compared with the L4 stage (with the exception of *mtl-2*) and provides further support that the L3 stage is transcriptionally more responsive to Cd than the L4 stage, at least for the majority of genes investigated.

The observation that *mtl-1, mtl-2*, and *cdr-1* genes are induced by Cd is not new and has been reported by others.^19,40^ This independent validation of the RNAseq results, therefore, provides confidence in the reproducibility of the RNAseq output and supported previous findings.^19,41^ However, it should be noted that the respective experimental designs differed. Namely, Cui and colleagues^19^ dosed worms via liquid cultures, used mixed-stage populations and significantly higher concentrations of Cd (100 µM), while Dölling^41^ utilized adult stage, low concentrations of Cd (0.1 and 10 µM) and a different source of bacteria (*HT115*). Therefore, these variations may limit the value of direct comparison.

### Functional enrichment analysis of the cadmium exposome

The exposure to Cd revealed changes in the global gene expression pattern. The presence of functional enrichment was explored by means of the Worm Enricher database (Tables 2 and 3) and Cytoscape (Supplementary Figs. 2 and 3).^42,43^ This analysis offered an overview of the pathways that are impacted by the treatment, which separated into two broad groups.

**Table 2.** Functional enrichment analysis of significant differentially expressed genes in *C. elegans*.

A	
GO term (biological process)	*P*-value
Response to cadmium ion (GO:0046686)	0.00008
Response to metal ion (GO:0010038)	0.007
Proteolysis (GO:0006508)	0.02
Cellular iron ion homeostasis (GO:0006879)	0.02
Positive regulation of autophagy (GO:0010508)	0.02
Acylglycerol catabolic process (GO:0046464)	0.02
Ammonium transmembrane transport (GO:0072488)	0.03
**GO term (molecular function)**	***P*-value**
Lipase activity (GO:0016298)	0.006
Hydrolase activity (GO:0016811)	0.01
Copper ion binding (GO:0005507)	0.03
Ferrous iron binding (GO:0008198)	0.03
Transition metal ion binding (GO:0046914)	0.03
Ammonium transmembrane transporter activity (GO:0008519)	0.04
Dipeptidyl-peptidase activity (GO:0008239)	0.05
**B**	
**GO term (biological process)**	***P*-value**
Collagen and cuticulin-based cuticle development (GO:0040002)	1.12 E-08
Cuticle development involved in moulting cycle (GO:0042338)	1.4 E-08
Protein auto-processing (GO:0016540)	0.02
Metabolic process (GO:0006505)	0.006
Membrane lipid metabolic process (GO:0006643)	0.02
Pyrimidine nucleobase metabolic process (GO:0006206)	0.02
Sphingolipid catabolic process (GO:0030149)	0.03
Extracellular matrix organization (GO:0030198)	0.005
Biosynthetic process (GO:0006506)	0.009
Phosphatidylinositol biosynthetic process (GO:0006661)	0.01
Glycolipid biosynthetic process (GO:0009247)	0.01
**GO term (molecular function)**	***P*-value**
Metallo-carboxypeptidase activity (GO:0004181)	0.000006
Carboxypeptidase activity (GO:0004180)	0.0002
Metallo-exopeptidase activity (GO:0008235)	0.0003
Carbon-nitrogen ligase activity (GO:0016884)	0.02
Sugar transmembrane transporter activity (GO:0051119)	0.02
Phspholipase activity (GO:0004620)	0.03

In total, 91 genes were significantly up-regulated (**A**) and 684 significantly down-regulated (**B**) in *C. elegans* exposed to 20 µM Cd from L1 to L3 stage (cut-off of ≥ 2 & ≤ −2 fold change and a *P*-value ≤ 0.05). The analysis was conducted with WormEnrichr. No significantly changing cellular component GO terms were identified. The respective GO terms and the *P*-values are indicated.

**Table 3.** Functional enrichment analysis of significant differentially expressed genes in *C. elegans*.

A	
GO term (biological process)	*P*-value
Response to cadmium ion (GO:0046686)	0.0003
Response to metal ion (GO:0010038)	0.01
Asparagine metabolic process (GO:0006528)	0.03
Positive regulation of autophagy (GO:0010508)	0.04
Acylglycerol catabolic process (GO:0046464)	0.04
Amino-acid betaine metabolic process (GO:0006577)	0.04
Aspartate family amino acid biosynthetic process (GO:0009067)	0.05
**GO term (molecular function)**	***P*-value**
Copper ion binding (GO:0005507)	0.04
Carnitine O-palmitoyltransferase activity (GO:0004095)	0.05
O-palmitoyltransferase activity (GO:0016416)	0.05
Carbon-nitrogen ligase activity, with glutamine as amido-N-donor (GO:0016884)	0.05
**B**	
**GO term (biological process)**	***P*-value**
Nucleotide-sugar metabolic process (GO:0009225)	0.01
Dosage compensation by hypoactivation of the X chromosome (GO:0042464)	0.02
Establishment or maintenance of apical/basal cell polarity (GO:0035088)	0.02
Nucleotide-sugar biosynthetic process (GO:0009226)	0.02
Dosage compensation (GO:0007549)	0.02
Response to gamma radiation (GO:0010332)	0.02
Maintenance of epithelial cell apical/basal polarity (GO:0045197)	0.02
3ʹ-UTR-mediated mRNA destabilization (GO:0061158)	0.03
mRNA destabilization (GO:0061157)	0.03
Phospholipid metabolic process (GO:0006644)	0.04
Response to ionizing radiation (GO:0010212)	0.05
**GO term (molecular function)**	***P*-value**
Phospholipase activity (GO:0004620)	0.03
Hydro-lyase activity (GO:0016836)	0.04
Lipase activity (GO:0016298)	0.04

In total, 141 genes were significantly up-regulated (**A**) and 38 significantly down-regulated (**B**) in *C. elegans* exposed to 20 µM Cd from L1 to L4 stage (cut-off of ≥ 2 & ≤ −2 fold change and a *P*-value ≤ 0.05). The analysis was conducted with WormEnrichr. No significantly changing cellular component GO terms were identified. The respective GO terms and the *P*-values are indicated.

Firstly, pathways that affected both L3 and L4 staged worms entailed overrepresented genes linked to stress response, defence mechanisms, lipid metabolism, and protein homeostasis. The functional enrichment of the most down-regulated genes was primarily correlated with growth, affecting functions related to cuticle, collagen, protein, and lipid metabolism. These pathways have in the past been linked to the stress response,^44,45^ e.g. several authors reported the association between stress and defence mechanism and the mitogen-activated protein kinase (MAPK) acting as the mediator.^19,44,46,47^ Noteworthy is the observation that the response cascade was significantly more complex and impacting in worms sampled at the L3 stage compared with the L4 stage. This clearly highlights that, despite the shorter exposure time, L3-staged worms seem to be more dynamic in their response to the Cd exposure.

Secondly, the differential expression of some transcripts was stage-specific; e.g. Heat shock protein (*hsp-70* and *hsp-16.2)* previously linked to Cd toxicity, were up-regulated at the L4 stage only.^48^ Findings were also reported by Dölling^41^ who described a similar result, suggesting that a more extended period of exposure might be required to trigger the induction of *hsp* genes. Likewise, numerous genes and pathways overrepresented in the exposed L3 stage were linked to oxidative stress, growth, and development. Only some of these differentially regulated transcripts are well characterized, making it challenging to associate them with specific functions and phenotypes; therefore, only a sub-section of genes can be highlighted. For instance, glutathione S-transferases (*gst-18* and *gst-19*), which are known to be involved in xenobiotic detoxification, were up-regulated at the L3 stage, along with other differentially expressed transcripts, including members of the collagen *(col)*, activated in blocked unfolded protein response *(abu)*, cuticlin-like *(cutl)*, and lipase-like *(lipl*) gene families. While other multigene families were downregulated, including the patched-related (*ptr*) family and hedgehog-related (*hhr*) genes, the latter include members of the warthog *(wrt)*, ground-like *(grl*), and groundhog *(grd)* genes. The *HHR* family is well known for their essential role in cuticle component secretion, mainly in hypodermal development,^49–51^ in addition to their cysteine (Cys) richness, which might suggest their involvement in promoting resistance to toxicants. Furthermore, the *nas* (astacin) family, which function as metalloendopeptidases,^52,53^ were also found to be down-regulated at the L3 stage. The down-regulation of these genes might explain the elevation of *mtl-1* expression in L3 as they might play a role in the detoxification of Cd. This is supported by the observation that the expression of *mtl-1* diminished at the L4 stage (i.e. following an extended exposure period), which might suggest a level of adaptation or the action of other genes involved in Cd detoxification that are activated at the L4 stage but not the L3 stage. A potential other reason for the observed variation in *mtl-1* expression is the difference in body size of L3 and L4 larvae. It is well established that the metabolic rate of smaller organisms is higher than in their larger counterparts, and thus it is conceivable that the uptake of Cd varied in L3 and L4 stages due to differences in metabolic rates. This notion is supported by the observation regarding stage-specific sensitivities in the reproductive performance of Cd-exposed *C.elegans* and some aquatic organisms (e.g. Zn toxicity and effects on size in *Daphnia* sp.).^27,54^

To discern stage-specific differences (in the absence of Cd toxicosis), we identified genes that were significantly expressed in unexposed L3 vs. L4. This identified ∼9000 differentially expressed genes, and functional enrichment analysis of the top 200 up-down-regulated genes were probed further in terms of biological process GO ontology identifiers. Overall, these genes were attributed to larva development in both stages and associated with anatomic features, e.g. IL sheath cell (WBbt:0008412) development in L3, whilst in L4 these genes are mostly linked to reproductive system growth e.g. spermatheca (WBbt:0005319) formation (Supplementary Tables 1 and 2). These transcripts are therefore linked to differences in stage/development and are likely independent of the identified responses upon a Cd challenge.

### *T08G5.1* expression

The RNAseq experiment revealed that the highest expressed transcript at the L4 stage and the second highest at the L3 stage was *T08G5.1*, a gene located immediately upstream of *mtl-2*. This indicates that the hitherto uncharacterized *T08G5.1* might be involved in the process driving the response to detoxify Cd.

**Fig. 2.**
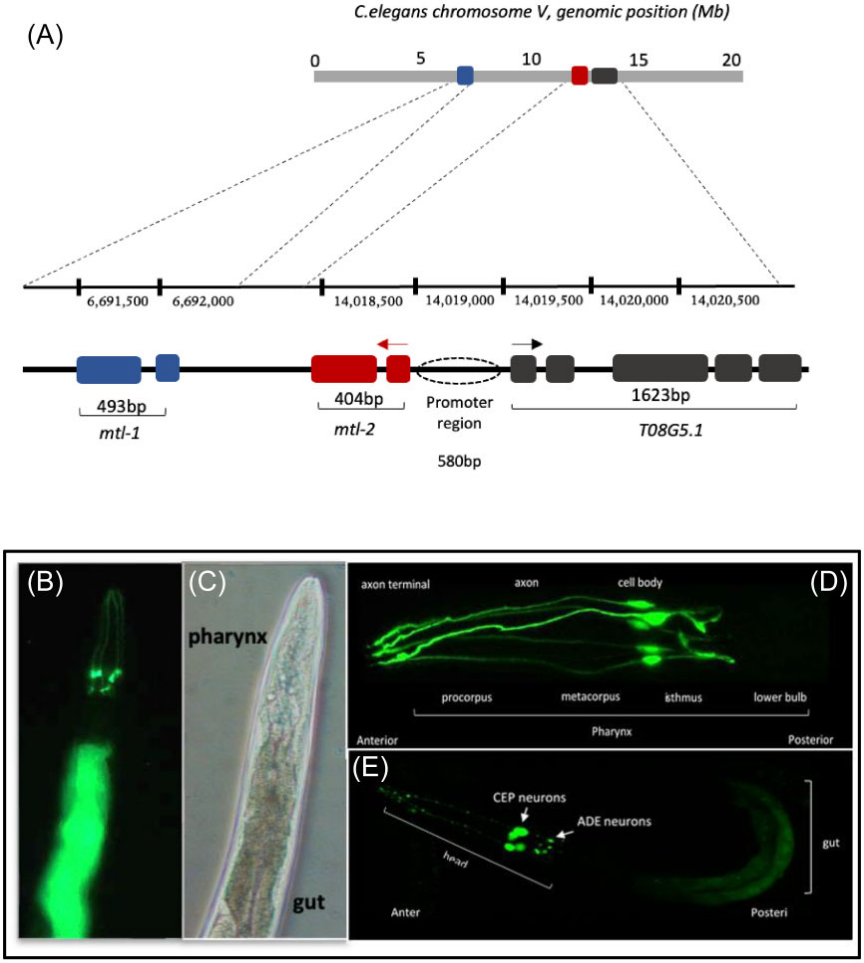
*T08G5.1* genomic location and expression localization in *C.elegans*. A schematic representation of *C. elegans* chromosome V displaying the location of the *mtl-1, mtl-2*, and *T08G5.1*. Note, *T08G5.1* is upstream of *mtl-2* with a shared promoter whereas *mtl-1* is located some 7 000,000 bp downstream (adapted from https://wormbase.org/) (**A**). The *T08G5.1* promoter was tagged, in frame, to GFP and the construct was microinjected into the gonads of worms to generate an extrachromosomal array. The worms were exposed to 20 µM Cd to determine the location of the gene expression, namely the gut and the neurosensory cells surrounding the pharynx. The images were taken with a 20 × objective to visualize the expression across larger sections of the body area of L4 stage (**B** and **C**). The neurosensory cells, which we predict to be CEP/ADE neurons at the lower part of the pharynx, were inspected in adult worms using a 60 × objective (**D**). The GFP signal of a developmentally arrested transgenic L1 worm following the exposure to 20 µM Cd was captured using a 60 × objective (**E**). Images were taken either by a Nikon inverted fluorescence microscope with blue laser scanning fluorescence (*λ*ex = 450–490 nm) to excite the GFP (**B** and **C**) or with an A1R confocal Nikon microscope (**D** and **E**).

**Fig. 3.**
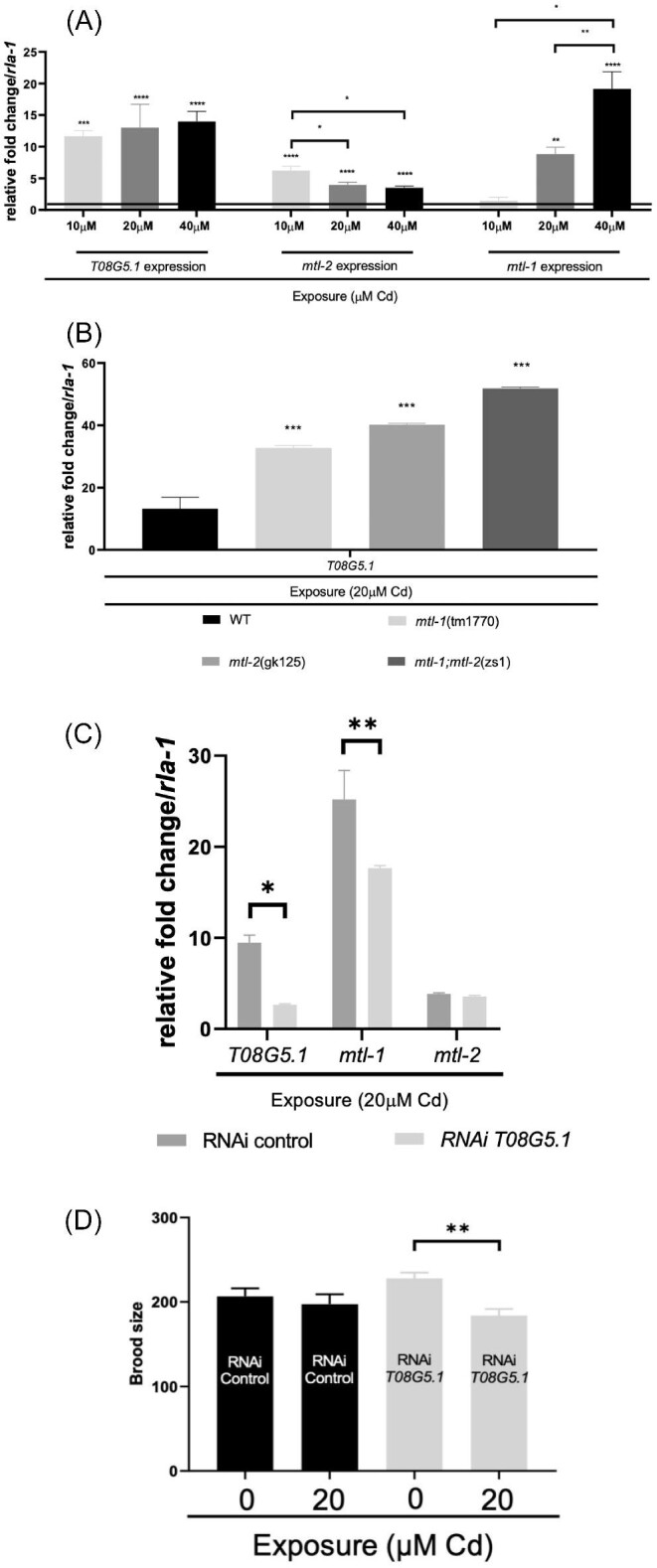
The expression fold change of *T08G5.1, mtl-1*, and *mtl-2* in WT *C. elegans* exposed to 10 µM, 20 µM, and 40 µM Cd for 27 h (from synchronized L1 stage–L3 stage) compared with unexposed controls (black horizontal baseline) (**A**). The expression of *T08G5.1* in worms exposed to 20 µM Cd in WT, in *mtl-1*(tm1770) and *mtl-2*(gk125) single knockout strains and the *mtl-1; mtl-2*(zs1) metallothionein double knockout. The data were expressed as relative fold change compared with the respective unexposed strains (**B**). The expression of *T08G5.1* was knocked down in WT worms by means of RNAi, the expression of *T08G5.1, mtl-1*, and *mtl-2* were quantified by qPCR, then normalized against the housekeeping gene *rla-1*. Three biological replicates were used per condition, each analysed via three technical repeats (**C**). Changes in brood size upon RNAi-mediated knockdown of *T08G5.1* were evaluated in worms exposed to Cd for 47 h (**D**). Note RNAi requires *E.coli* HT115 (rather than OP50), thus baselines between WT and RNAi differ. A *T*-test was applied to identify significant differences between treated and untreated groups, a one-way ANOVA was utilized as well to show significance between exposed groups followed by Tukey's multiple comparisons (**A, B**, and **D**), and a two-way ANOVA was utilized to identify statistically significant differences between exposed groups followed by a Sidak's multiple comparison test (**C**). (*P ≤ 0.05, **P ≤ 0.01, and ****P ≤ 0.0001). Error bars denote ± SEM.

*T08G5.1* is expressed in the gut cells in the posterior region, as well as in the neuronal cells of the head region (Fig. 2-B-E), most likely CEP/ADE neurons.^55–57^ The expression of *T08G5.1* is strongly induced in L3 worms (exposed to Cd for 27 h), in a dose-responsive manner (Supplementary Fig. 4). Despite *T08G5.1* and *mtl-2* sharing the same promoter and *mtl-1* positioned some 7Mb upstream on chromosome V (Fig. 2-A), it is surprising to note that the expression dynamics of *T08G5.1* resemble *mtl-1* more than *mtl-2* (Fig. 3-A). The qPCR validated the key outcome from the RNAseq in this study and others^19^ confirming that the expression of *T08G5.1* is highly induced following the exposure to Cd. However, Cd is not the sole driver of *T08G5.1* expression as other xenobiotics seem to induce its expression as well (albeit at lower levels), including perchlorate^58^ and benzo[*a*]pyrene,^59^ which induced *T08G5.1* 1.3 and 1.9 fold, respectively. T08G5.1 is an uncharacterized protein made up of 29 Arg (12%) and 22 Pro (9.1%) residues. It is larger than MTLs (T08G5.1: 241aa vs MTL-1: 75aa and MTL-2: 63aa) and contains only few Cys residues (T08G5.1: 3 Cys vs MTL-1: 19 Cys and MTL-2: 18 Cys) (Supplementary Table 3).^15^

The observation that Cd exposure resulted in an elevated expression of *T08G5.1*, not dissimilar to the expression of *mtl-1*, plus the fact that it shares a common promoter with *mtl-2* suggests that these three genes might be functionally connected. *T08G5.1* expression was therefore explored in *mtl-1* and *mtl-2* single and double mutants, which revealed an increase in *T08G5.1* expression (32.6-fold in *mtl-1*(tm1770), 40.1-fold in *mtl-2*(gk125) and 51.8-fold in *mtl-1;mtl-2*(zs1)), possibly indicating that *T08G5.1* can compensate for the absence of MTLs (Fig. 3-B). In addition, the knockdown of *T08G5.1* by RNAi affected the expression of *mtl-1* to some extent (reducing the exposure mediated induction from 25 in WT to 17.6 fold upon RNAi of *T08G5.1*), indicating that *mtl-1* and *T08G5.1* might be involved in the same or similar stress responsive pathways (Fig. 3-C). Given that *T08G5.1* was up-regulated upon exposure to different toxicants and the circumstantial link to MTLs, suggests that *T08G5.1* may indeed act to protect the organism from the direct consequences of Cd toxicosis or a more indirect build-up of oxidative stress.^60,61^ To substantiate this notion, further work should be conducted on *T08G5.1* to dissect the difference and possible interplay between Cd metabolism/detoxification and the handling of oxidative stress.

We further examined the impact of T08G5.1 knockdown by RNAi on reproductive output (brood size). The analysis revealed that, in the presence of Cd exposure, the number of offspring produced was significantly reduced when T08G5.1 was knocked down. In contrast, under control conditions (in the absence of a Cd challenge), reproductive performance was statistically indistinguishable between unexposed and exposed RNAi (Fig. 3-D). It should be noted that a direct comparison between WT and RNAi control worms is challenging due to the differences in diet (standard *E.coli* OP50 vs the richer HT115, respectively). *T08G5.1* is expressed in CEP and ADE neuronal cells, which are categorised as dopaminergic neurons.^62^ Cd accumulation can reduce dopamine levels, which, in turn, can induce neurochemical disturbances in the organism.^63,64^ In *C. elegans*, dopamine controls the response to environmental stimuli, and has been used to investigate neurodegeneration and Parkinson's disease.^65–68^

To gain an understanding regarding the potential function of this gene, the shared promoter positioned between *mtl-2* and *T08G5.1* was bioinformatically scanned for putative transcription factor binding sites using JASPAR 2020 software.^69^ Despite the absence of distinct metal response elements (MREs), several putative candidates were identified, including CHE-1 and SMA-4 that were previously linked to the response to metal ions within neurons cells^16^ and thus should be investigated further (Supplementary Fig. 5).

## Conclusions

This study reveals that *C. elegans* raised in the presence of Cd are characterized by stage-specific transcriptional responses. The transcriptome of the L3 larva seemed to be more affected than L4 worms, as demonstrated by the difference in statistically significant changing transcripts and the magnitude of induction in the respective stages. This finding can be explained by two mutually non-exclusive hypotheses: (1) The induction of key detoxification mechanisms may reduce the toxic impact over time; namely, the worm adapts to the challenge; (2) Although most organs are fully developed at both stages, some fundamental differences prevail. For example, the L3 stage lacks a reproductive system that develops at the L4 stage. The presence or absence of reproductive organs might explain some of the differences observed in the stage-specific responses. The observed stage-specific differences highlight that any assay that assesses the impact of environmental toxicants via molecular genetic probes should not neglect stage-specific effects.

Evidently, Cd toxicity is not limited to the expression of *MTL*s; other (uncharacterized) genes are also involved. Based on the transcriptional analysis, *T08G5.1* is linked to the defence process against metal stress and/or possibly oxidative stress. The expression of *mtl-1* seems to be modulated by the absence of *T08G5.1*. The interdependence of *T08G5.1* and *mtl-2* is less pronounced despite them sharing the same promoter region on chromosome V (where *T08G5.1* is positioned on the antisense strand and *mtl-2* on the sense strand, which is positioned upstream of *T08G5.1*. with 580 bp of shared promoter sequence). Taken together, *T08G5.1* seems to be an interesting new player in the growing field of metallomics.

## Supplementary Material

mfae016_Supplemental_File

## Data Availability

The data underlying this article will be shared on reasonable request to the corresponding author.
