# Supplementary material for: Stage-specific exposure of Caenorhabditis elegans to cadmium identifies unique transcriptomic response cascades and an uncharacterized cadmium responsive transcript
Source: Metallomics. 2024 Mar 28;16(5):mfae016. doi: 10.1093/mtomcs/mfae016 (PMC11066929; doi:10.1093/mtomcs/mfae016)
Supplement: mfae016_Supplemental_File [file mfae016_supplemental_file.pdf]

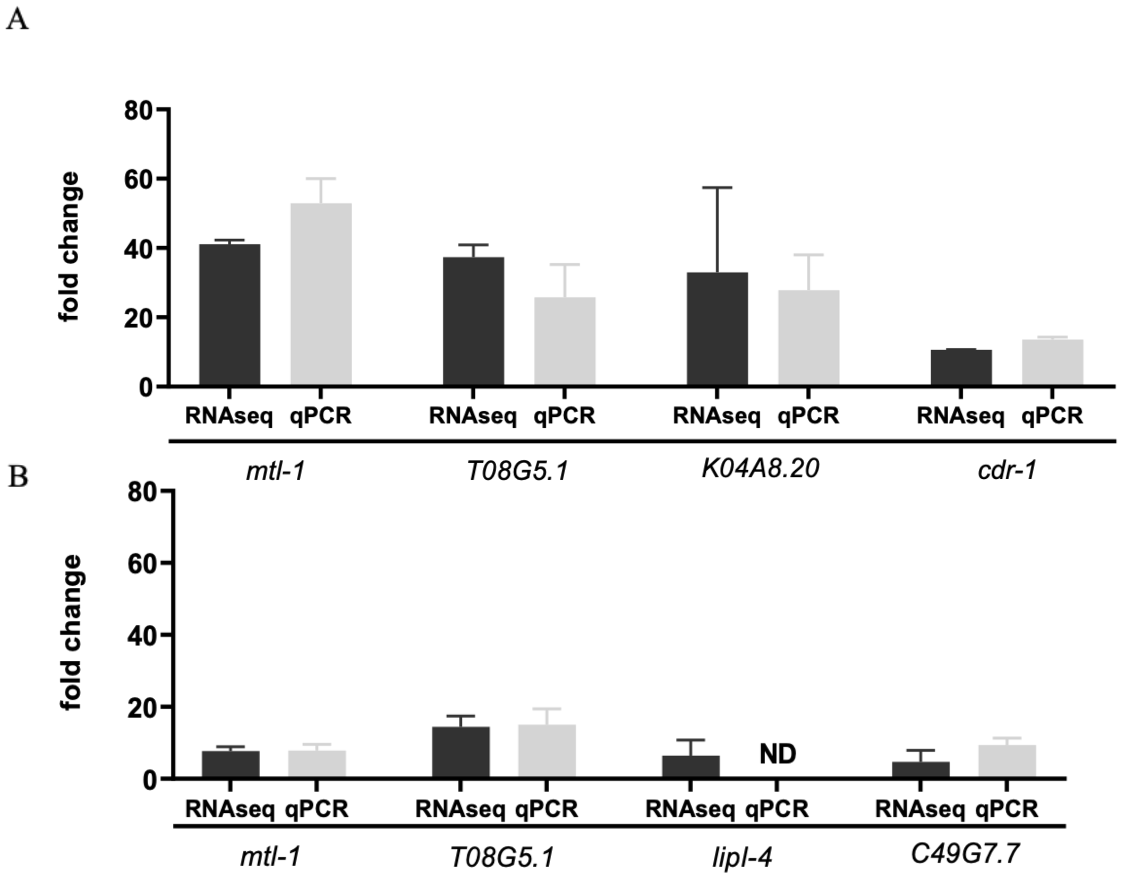

Supplementary, figure 1: Data validation of RNAseq results of differentially expressed transcripts. The highest expressed genes at L3 (A) and L4 (B) stage identified by RNAseq were chosen and re-analyzed by means of the qPCR. In total, 7 out of the 8 qPCR-based results aligned well with the RNAseq results, but one qPCR yielded undetectable results. The qPCR data represent the mean ± SEM of 3 biological replicates. ND = not detectable.

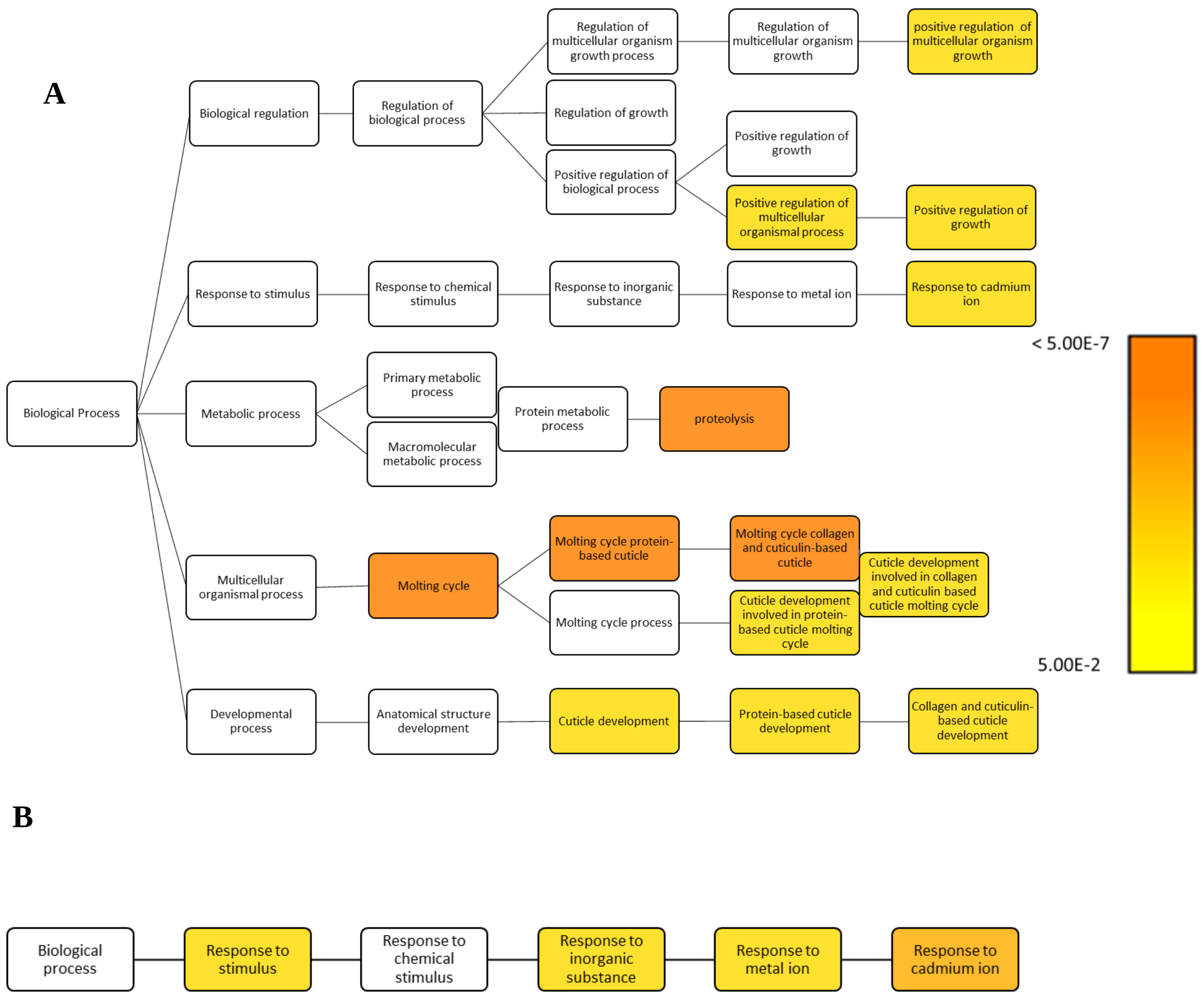

Supplementary, figure 2: Statistically significant overrepresented biological process GO Terms enriched in worms exposed to 20 µM Cd from L1 to L3 (A) or from L1 to L4 (B) stage. The gene list generated was based on a cut-off of ≥ 2 & ≤ -2 fold change and a p-value ≤ 0.05, resulting in 775 transcripts for L3 and 179 transcripts for L4. The legend bar indicates the statistically significant enriched functions. The analysis was conducted with Cytoscape using the BiNGO tool.

A

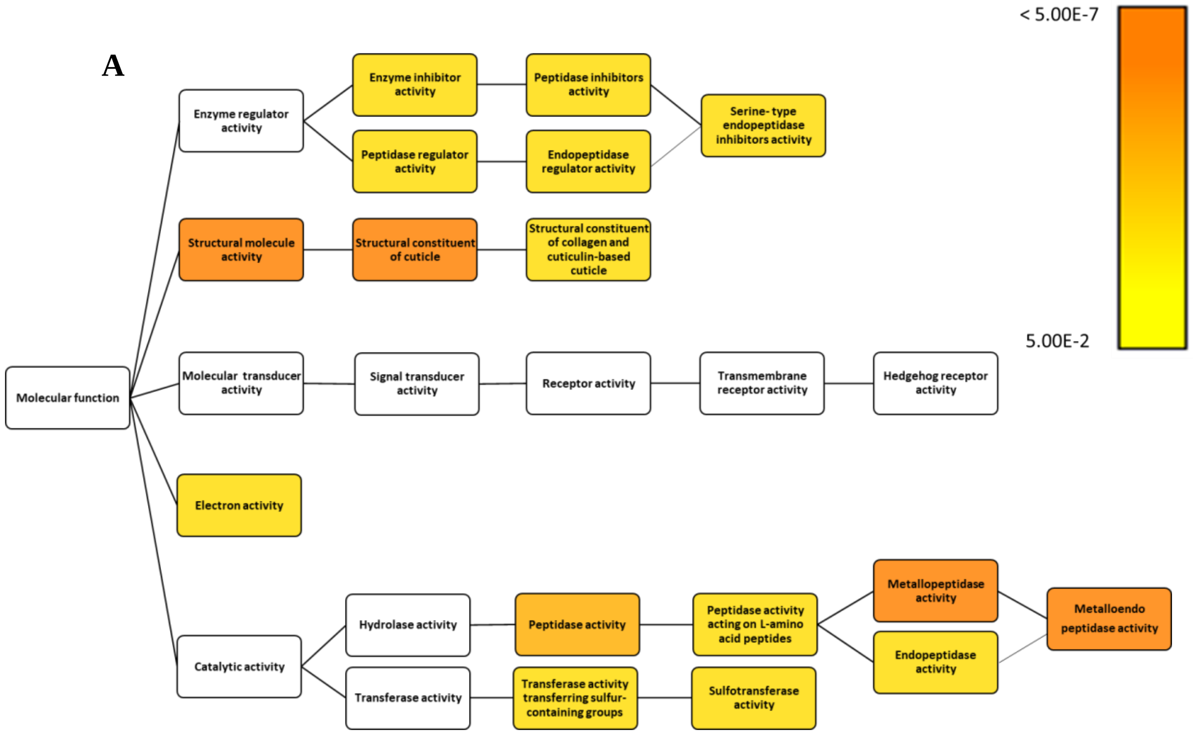

B

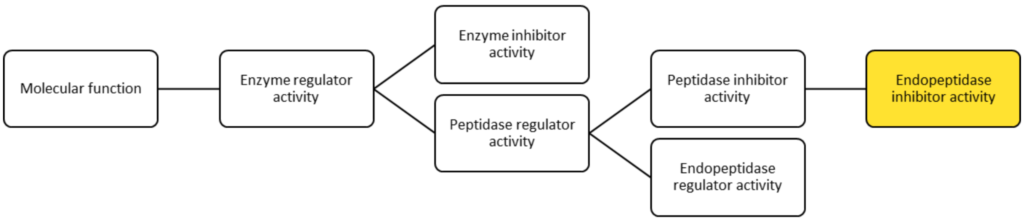

Supplementary, figure 3: Significantly overrepresented molecular function GO Terms enriched in worms exposed to 20 µM Cd from L1 to L3 (A) or L1 to L4 (B) stage. The gene list generated was based on a cut-off of ≥ 2 & ≤ -2 fold change and a p-value ≤ 0.05, resulting in 775 transcripts for L3 and 179 transcripts for L4. The legend bar indicates the statistically significant enriched functions. The analysis was conducted with Cytoscape using the BiNGO tool.

L3 vs L4 (unexposed conditions)			
**Upregulated genes**			
	**Gene**	**Log2 Fold Change**	**P-value**
1	*msp-65*	14.21	5.6E-33
2	*Y57G11B.5*	13.63	5.5E-30
3	*R04D3.3*	13.61	4.8E-30
4	*spch-3*	13.61	2.2E-30
5	*ZK813.1*	13.37	3.4E-29
6	*C33F10.1*	13.37	2.0E-29
7	*F58A6.9*	13.19	1.6E-28
8	*W04A4.2*	13.17	1.4E-28
9	*Y69E1A.1*	13.13	1.7E-28
10	*ZK938.1*	13.12	2.4E-28
11	*msp-57*	13.07	5.5E-37
12	*col-135*	12.92	4.3E-27
13	*C30G7.3*	12.92	1.6E-27
14	*msp-78*	12.90	1.4E-27
15	*D1086.7*	12.89	3.0E-27
**Downregulated genes**			
	**Gene**	**Log2 Fold Change**	**P-value**
1	*Y17D7C.2*	-8.49	1.25E-11
2	*F17B5.6*	-8.20	3.41E-10
3	*tba-8*	-7.75	3.61E-49
4	*C06A1.9*	-7.59	0.01
5	*nhr-265*	-7.42	2.55E-08
6	*C13A2.3*	-7.07	9.03E-18
7	*C27A7.2*	-6.77	6.61E-11
8	*F32H2.6*	-6.62	2.98E-09
9	*ZK180.18*	-6.55	7.64E-07
10	*col-54*	-6.43	9.97E-92
11	*lips-6*	-6.37	2.26E-27
12	*col-41*	-6.35	2.84E-109
13	*gst-18*	-6.33	5.01E-06
14	*ins-35*	-6.30	1.07E-91
15	*C36B7.4*	-6.27	1.38E-27

Supplementary, table 1: Differential gene expression of unexposed *C. elegans* stage L3 vs L4. Nematodes were cultured on NGM plates supplemented with OP50 from L1 to L3 stage (27 h) and from L1 to L4 stage (47 h) then processed for RNAseq analysis. The top 15 significant up/down regulated genes with a p-value of ≤ 0.05 are listed.

A	GO term (upregulated genes)	P value
1	extracellular matrix organization (GO:0030198)	0.001
2	protein phosphorylation (GO:0006468)	0.02
3	peptidyl-serine phosphorylation (GO:0018105)	0.02
4	peptidyl-serine modification (GO:0018209)	0.02
5	medium-chain fatty acid metabolic process (GO:0051791)	0.02
6	G2/M transition of mitotic cell cycle (GO:0000086)	0.02
7	medium-chain fatty acid catabolic process (GO:0051793)	0.02
8	cell cycle G2/M phase transition (GO:0044839)	0.02
9	organelle disassembly (GO:1903008)	0.03
10	ribonucleoprotein complex disassembly (GO:0032988)	0.03
11	DNA replication (GO:0043137)	0.03
12	positive regulation of meiosis I (GO:0060903)	0.04
13	positive regulation of RNA polymerase II (GO:0045899)	0.04
14	regulation of plasma membrane bounded cell projection assembly (GO:0120032)	0.04
15	positive regulation of meiotic cell cycle phase transition (GO:1901995)	0.04
16	positive regulation of transcription initiation from RNA polymerase II promoter (GO:0060261)	0.04
17	regulation of RNA polymerase II transcriptional preinitiation complex assembly (GO:0045898)	0.04
18	DNA replication, Okazaki fragment processing (GO:0033567)	0.04
19	RNA phosphodiester bond hydrolysis, endonucleolytic (GO:0090502)	0.05
20	phosphate ion transport (GO:0006817)	0.05
21	phosphate ion transmembrane transport (GO:0035435)	0.05
22	mitotic cell cycle phase transition (GO:0044772)	0.05

B	GO term (downregulated genes)	P value
1	neuromuscular junction development (GO:0007528)	0.003
2	cuticle development involved in collagen and molting cycle (GO:0042338)	0.005
3	extracellular matrix organization (GO:0030198)	0.01
4	post-embryonic body morphogenesis (GO:0040032)	0.01
5	collagen and cuticulin-based cuticle development (GO:0040002)	0.01
6	epithelium development (GO:0060429)	0.02
7	response to reactive oxygen species (GO:0000302)	0.02
8	gamma-aminobutyric acid receptor clustering (GO:0097112)	0.05
9	neurotransmitter-gated ion channel clustering (GO:0072578)	0.05
10	regulation of defense response to bacterium, incompatible interaction (GO:1902477)	0.05
11	protein localization to actomyosin contractile ring (GO:1990179)	0.05
12	GTP metabolic process (GO:0046039)	0.05
13	Mo-molybdopterin cofactor biosynthetic process (GO:0006777)	0.05
14	positive regulation of eating behavior (GO:1904000)	0.05
15	Mo-molybdopterin cofactor metabolic process (GO:0019720)	0.05
16	regulation of multicellular organism growth (GO:0040014)	0.05

Supplementary, table 2: Functional enrichment analysis of the significantly changing transcripts in unexposed *C. elegans* stage L3 vs L4. Nematodes were cultured on NGM plates supplemented with OP50 for 27 h (L1 to L3 stage) and 47 h (L1 to L4 stage). The top 200 significant up/down regulated genes were further analyzed by means of WormEnrichr focussing on the GO term Biological Process. The respective GO terms and the p-values are indicated.

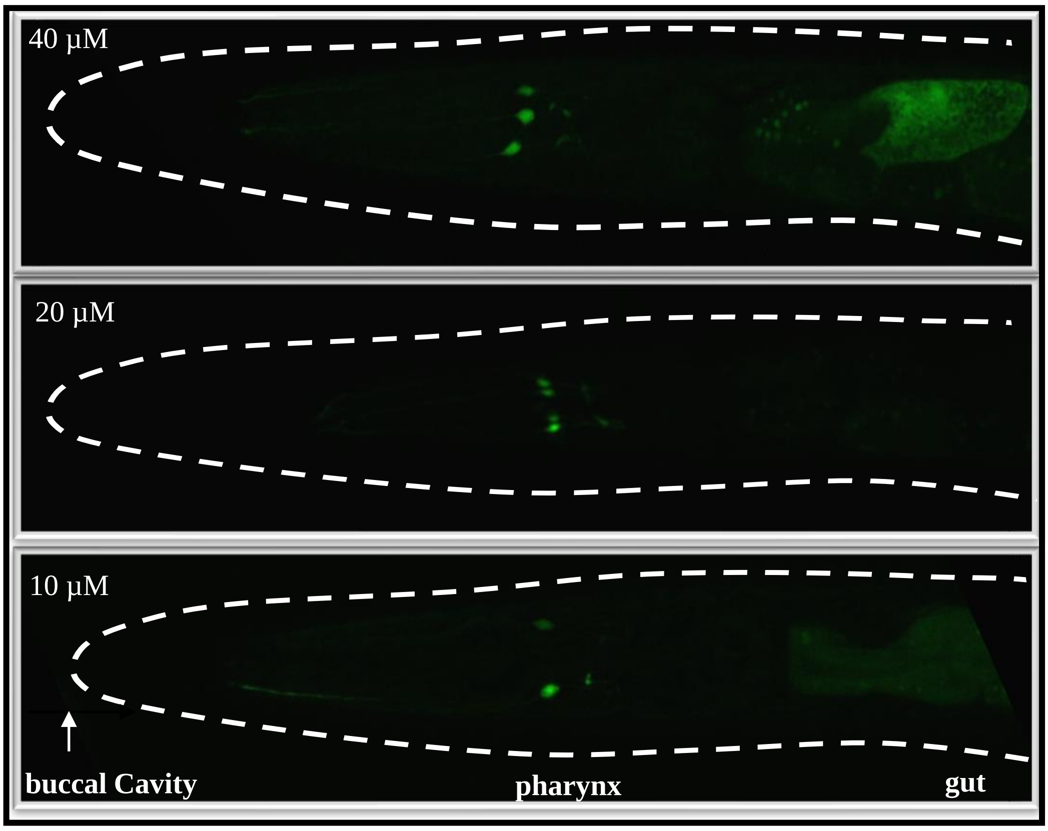

Supplementary, figure 4: The transgenic *T08G5.1*::GFP worms were exposed for 72h to 10, 20, 40 µM Cd. A cadmium dose-dependent increase in fluorescent signal was observed in the gut and the neurosensory cells at the lower part of the metacorpus of the pharynx. The images were taken applying the same settings of the blue laser by means of a A1R confocal Nikon microscope using a 20✖ objective at *λex* = 450-490 nm and analysed further by means of the ImageJ 1.49C software.

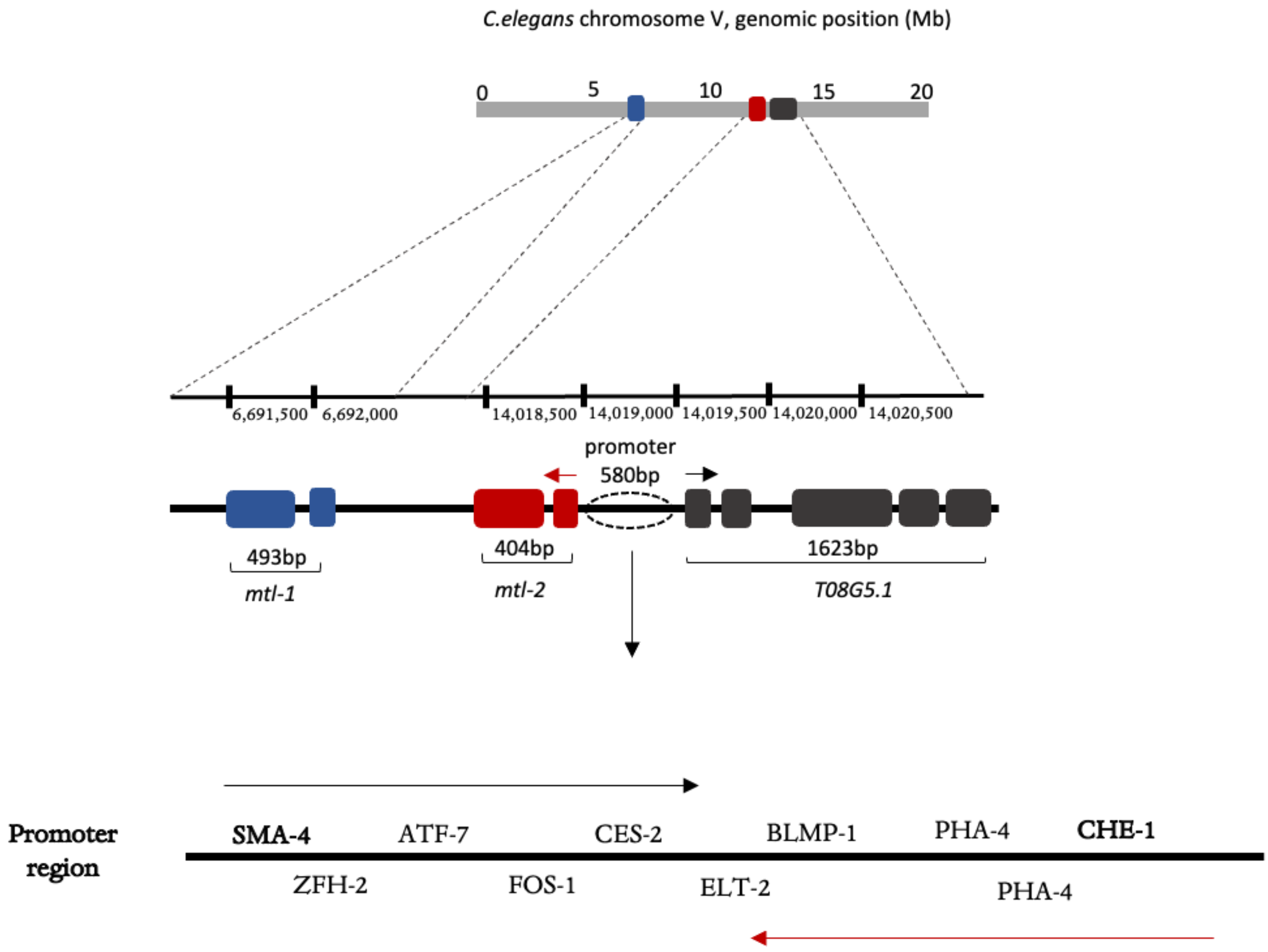

Supplementary, figure 5: A schematic representation of *C. elegans* chromosome V displaying the location of the *mtl-1*, *mtl-2* and *T08G5.1*. The promoter binding site was screened to predict transcription factor binding sites. Multiple elements were identified in the shared promoter region, with CHE-1 and SMA-4 promising candidates as both are involved in metal ions response and expressed in neuron cells (the transcription factors in this illustration were distributed randomly). Note, *T08G5.1* is upstream of *mtl-2* with a shared promoter whereas *mtl-1* is located some 7,000,000 bp downstream (adapted from https://wormbase.org/).

	T08G5.1		MTL-1		MTL-2	
AA	Number	%	Number	%	Number	%
Ala	7	2.9	8	10.7	7	11.1
Arg	29	12.0	-	-	-	-
Asn	12	5.0	2	2.7	4	6.3
Asp	15	6.2	5	6.7	4	6.3
Cys	3	1.2	19	25.3	18	28.6
Gln	10	4.1	5	6.7	3	4.8
Glu	13	5.4	6	8.0	3	4.8
Gly	8	3.3	6	8.0	4	6.3
His	11	4.6	4	5.3	1	1.6
Ile	16	6.6	-	-	-	-
Leu	8	3.3	-	-	-	-
Lys	20	8.3	13	17.3	8	12.7
Met	6	2.5	1	1.3	1	1.6
Phe	7	2.9	-	-	-	-
Pro	22	9.1	1	1.3	1	1.6
Ser	17	7.1	2	2.7	4	6.3
Thr	16	6.6	2	2.7	3	4.8
Trp	2	0.8	-	-	-	-
Tyr	6	2.5	1	1.3	1	1.6
Val	13	5.4	-	-	1	1.6

Supplementary, table 3: The composition of the T08G5.1 protein and the percentage of each amino acid is indicated in comparison to other cadmium-responsive proteins, namely MTL-1, and MTL-2, Note: T08G5.1 is arginine (Arg)/proline (Pro) rich. The sequence and amino acids component of the protein was retrieved from www.wormbase.org.
